# Kojyl Cinnamate Ester Derivatives Increase Adiponectin Expression and Stimulate Adiponectin-Induced Hair Growth Factors in Human Dermal Papilla Cells

**DOI:** 10.3390/ijms20081859

**Published:** 2019-04-15

**Authors:** Phil June Park, Eun-Gyung Cho

**Affiliations:** Basic Research & Innovation Division, R&D Unit, Amorepacific Corporation, 1920 Yonggu-daero, Giheung-gu, Yongin-si, Gyeonggi-do 17074, Korea; mosme@amorepacific.com

**Keywords:** adiponectin, adiponectin inducer, kojyl cinnamate ester derivative, adipogenesis, hair growth-related factor, human follicular dermal papilla cell

## Abstract

Adiponectin (APN), released mainly from adipose tissue, is a well-known homeostatic factor for regulating glucose levels, lipid metabolism, and insulin sensitivity. A recent study showed that human hair follicles express APN receptors and the presence of APN-mediated hair growth signaling, thereby suggesting that APN is a potent hair growth-promoting adipokine. Previously, kojyl cinnamate ester derivatives (KCEDs) were synthesized in our institute as new anti-aging or adiponectin-/adipogenesis-inducing compounds. Here, we tested the activity of these derivatives to induce endogenous APN secretion. Among the derivatives, KCED-1 and KCED-2 showed improved activity in inducing APN mRNA expression, secretion of APN protein, and adipogenesis in human subcutaneous fat cells (hSCFs) when compared with the effects of Seletinoid G, a verified APN inducer. When human follicular dermal papilla cells were treated with the culture supernatant of KCED-1- or KCED-2-treated hSCFs, the mRNA expression of APN-induced hair growth factors such as insulin-like growth factor, hepatocyte growth factor, and vascular endothelial growth factor was upregulated compared with that in the control. Taken together, our study shows that among kojyl cinnamate ester derivatives, KCED-1, KCED-2, as well as Seletinoid G are effective inducers of endogenous APN production in subcutaneous fat tissues, which may in turn contribute to the promotion of hair growth in the human scalp.

## 1. Introduction

Adipose tissue, an active metabolic and endocrine organ, plays important roles in physiological and pathological processes by secreting a variety of soluble factors [1]. Particularly, subcutaneous fat, a white adipose tissue beneath the skin dermis and the largest adipose tissue in the body, is involved in regulating body temperature and skin elasticity in normal states. However, its dysregulation is associated with abnormal states including obesity, which has impacts on skin physiology, skin manifestations, dermatologic diseases, and lipodystrophy [2,3,4], indicating the important functions of adipocytes or adipocyte-derived factors in skin pathophysiology.

Adiponectin (APN), along with leptin, is a key hormone that is exclusively released from white adipocytes including those in subcutaneous adipose tissue and is involved in regulating glucose levels, lipid metabolism, and insulin sensitivity [5,6]. Three types of receptor for APN have been verified: adiponectin receptor 1 (AdipoR1), AdipoR2, and T-cadherin. AdipoR1 and AdipoR2 are integral membrane proteins possessing seven-transmembrane domains; the N- and C-termini are internal and external, respectively. Both receptors bind to APNs, which exist in a trimer, a hexamer, and high-molecular weight 12- to 18-mer by combining via collagen domain at the N-terminus, and activate p38 MAPK, AMPK, and PPARα, thereby mediating APN-induced biological functions such as increased fatty acid oxidation, increased glucose uptake, and decreased gluconeogenesis [5,7]. T-cadherin is suggested to be important for APN-mediated cardioprotection [7]. Besides its roles in regulating energy metabolism, APN was recently reported to decrease ceramide and increase sphingosine-1 phosphate, thereby protecting from apoptosis [7,8], and to display in vitro hair growth-promoting effects on human hair follicles that express three types of APN receptors [9]. APN promotes hair shaft elongation in organ culture, and this effect is comparable to that of minoxidil, a representative drug for promoting hair growth. Although the stimulation of APN-associated hair growth signals via APN receptors has been suggested as a potential clinical strategy, synthetic APNs are expensive, thereby necessitating alternative methods.

Previously, we synthesized kojyl cinnamate ester derivatives (KCEDs) via the sequential reaction of kojic acid (KA) with thionyl chloride and then with 3,4-(methylenedioxy) cinnamic acid (CA), generating Seletinoid G (SG; Compound 4b) [10,11], which shows anti-aging activity and promotes APN production in adipose tissue-derived stem cells (ADSCs) [11,12]. Similar to SG, two derivatives (Compounds 4a and 4c) were verified to show improved activity for APN production during adipogenesis, importantly indicating that an α,β-unsaturated carbonyl ester structure and intact KA moiety in the derivatives are essential for promoting adipogenesis. Because of the insoluble nature of the three verified derivatives, we further screened new KCEDs that harbor an α,β-unsaturated carbonyl structure with KA moiety but were estimated to show high solubility. Finally, we selected two derivatives, KCED-1 and KCED-2, and in this study examined their potential to induce APN expression and adipogenesis using human subcutaneous fat cells (hSCFs). Furthermore, we investigated whether these two compounds could be used to promote APN-associated hair-growth signals in human follicular dermal papilla cells (hDPCs).

## 2. Results and Discussion

### 2.1. Selected Kojyl Cinnamate Ester Derivatives Show No Significant Effect On hSCF Viability

**Figure 1:**
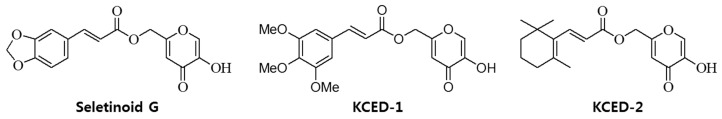
The structures of the kojyl cinnamate ester derivatives Seletinoid G, KCED-1, and KCED-2.

**Figure 2:**
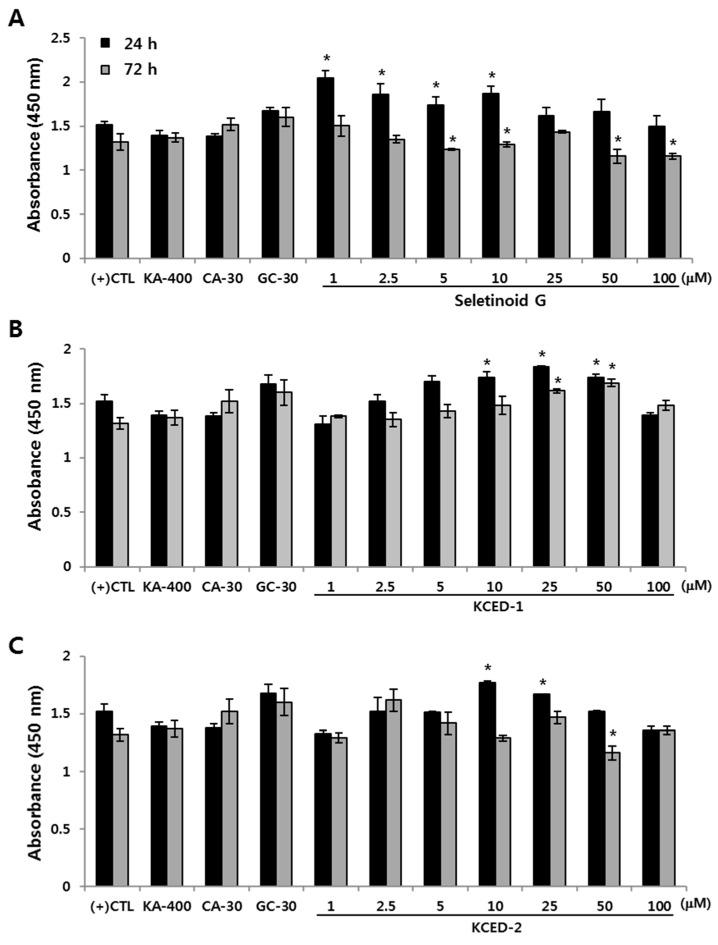
The effect of SG, KCED-1, and KCED-2 on cell viability of human subcutaneous fat cells (hSCFs). The hSCFs were treated with (**A**) SG, (**B**) KCED-1, and (**C**) KCED-2 for 24 or 72 h, after which the cell viability was analyzed. The data are presented as the mean ± SD (* *p* < 0.05). KA-400, kojic acid-400 μM; CA-30, cinnamic acid-30 μM; GC-30, glibenclamide-30 μM; CTL, control.

To determine the appropriate non-cytotoxic concentration of various compounds including SG and newly selected derivatives (KCED-1 and KCED-2) (Figure 1), hSCFs were treated with each compound and cell viability was measured at 24 and 72 h. KA and cinnamic acid (CA), which are structural moieties for KCEDs [10], and glibenclamide (GC), a well-known adiponectin inducer [13], were used as negative or positive controls. These control compounds did not cause severe changes in cell viability at tested concentrations (Figure 2). SG increased cell viability at concentrations ≤ 10 μM when treated for 24 h but decreased significantly at concentrations ≥ 5 μM at 72 h (Figure 2A). Compared with SG, KCED-1 and -2 increased cell viability at concentrations ≥ 10 μM at 24 h (Figure 2B,C). In particular, KCED-1 increased cell viability at concentrations of 25 μM and 50 μM even at 72 h, whereas SG and KCED-2 did not increase or decrease cell viability (Figure 2B,C), suggesting that KCED-1 and -2, and particularly KCED-1, are less toxic to the cells than SG. Although SG and KCED-2 showed significant cytotoxicity at some concentrations, according to ISO 10993-5, substances eliciting a cell viability above 80% are considered to be non-cytotoxic. Therefore, various concentrations (10 μM, 25 μM, and 50 μM) of these three compounds were applied for examining cellular effects.

### 2.2. Selected Kojyl Cinnamate Ester Derivatives Induce mRNA Expression of APN 

**Figure 3:**
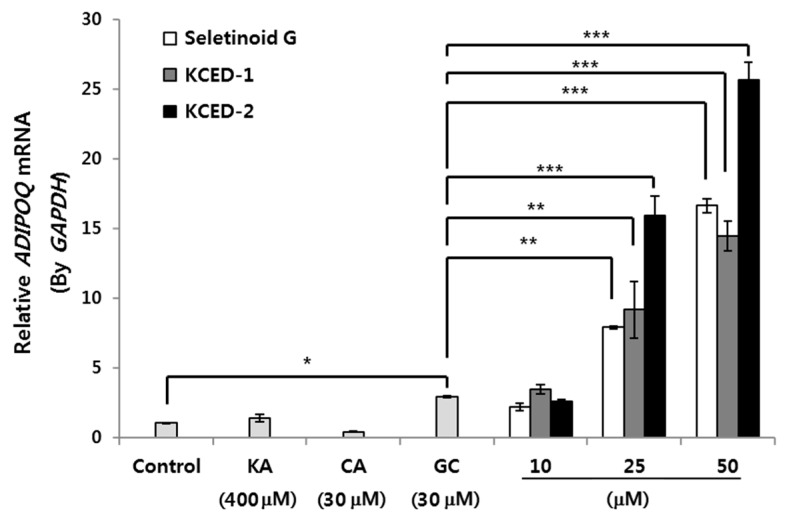
Upregulation of *ADIPOQ* mRNA expression by SG, KCED-1, and KCED-2 in hSCFs. The hSCFs were treated with the indicated concentration of each compound for 14 days, and the mRNA expression of *ADIPOQ* was analyzed by qRT-PCR. The data are presented as the mean ± SD (*n* = 3; * *p* < 0.05, ** *p* < 0.01, and *** *p* < 0.001). KA, kojic acid; CA, cinnamic acid; GC, glibenclamide.

It is known that APN is released into the medium when adipocytes mature by forming lipid droplets in the cytosol [11,14]. To validate whether newly selected KCED-1 and -2 are effective in APN production and secretion, we treated the differentiated hSCFs with the compounds and examined the mRNA expression levels of APN. SG has the ability to differentiate adipocytes and promote APN production [11]; therefore, we compared the activities of the selected compounds with that of SG. The mRNA expression of APN gene (*ADIPOQ*) in hSCFs was greatly upregulated by treatments with SG, KCED-1, or KCED-2 in a dose-dependent manner compared with that of GC, the positive control for APN induction (Figure 3). KA and CA did not induce the mRNA expression of *ADIPOQ* significantly, and KCED-2 showed the strongest effect on increasing *ADIPOQ* levels at concentrations of 25 μM and 50 μM.

### 2.3. Selected Kojyl Cinnamate Ester Derivatives Promote APN Secretion and Stimulate Adipogenesis in hSCFs

**Figure 4:**
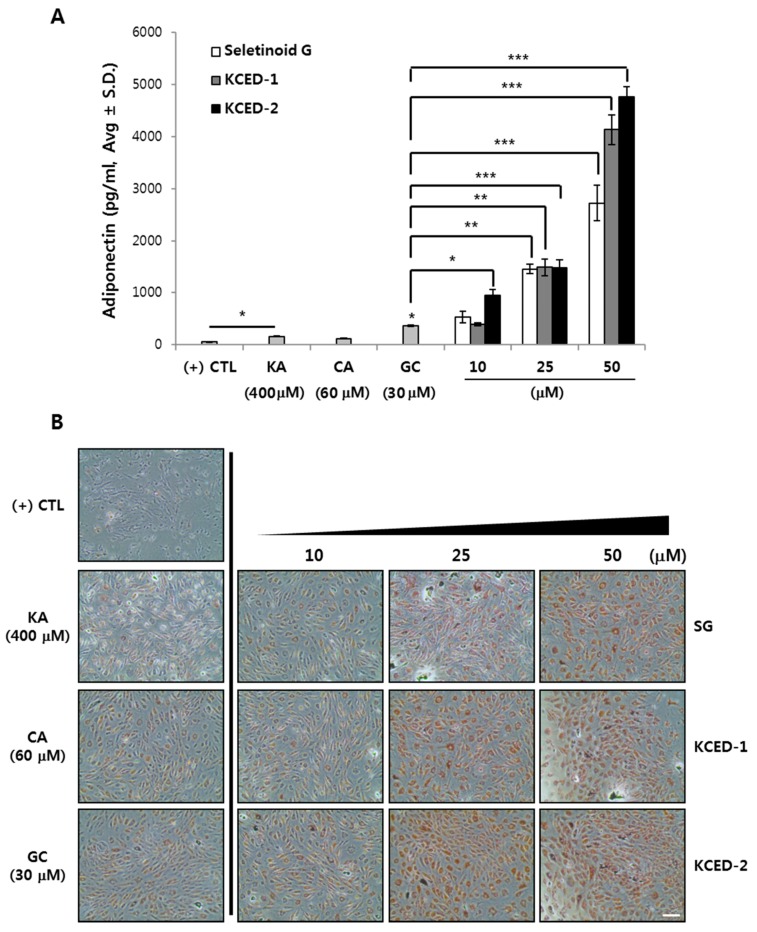
SG, KCED-1, and KCED-2 promote adiponectin secretion and stimulate adipogenesis in hSCFs. The hSCFs were pre-cultured for two days and differentiated for another 14 days using the differentiation medium supplemented with each compound. (**A**) The secreted APN levels were quantitatively determined using ELISA assay kit. The data are presented as the mean ± SD (*n* = 3; * *p* < 0.05, ** *p* < 0.01, and *** *p* < 0.001). (**B**) Lipids droplets were stained with Oil Red O dye. Scale bar, 200 μm.

Inspired by the dramatic increase in *ADIPOQ* mRNA levels, we determined the secreted protein levels of APN after treatments with SG, KCED-1, and KCED-2 during differentiation of hSCFs. Compared with the non-treated control, treatments with KA and GC, but not CA, increased the secretion of APN weakly but significantly (Figure 4A). Compared with GC, SG, KCED-1, and KCED-2 dramatically increased the APN secretion at concentrations of 25 μM and 50 μM. Consistent with the result in mRNA expression, KCED-2 treatment increased the APN secretion significantly at concentrations as low as 10 μM, which is one-third of the GC concentration, and showed the strongest effect on APN induction. APN is an essential factor in regulating lipid metabolism by inducing adipogenesis, fatty acid oxidation, and mitochondria biogenesis, thereby protecting obesity, insulin resistance, and type 2 diabetes [15,16,17]. We examined the degree of adipocyte differentiation by staining the lipid droplets formed after treatment with each compound. Compared with the non-treated control cells, where the lipid droplets were barely formed and stained, hSCFs treated with KA, CA, or GC showed weak but increased signals in Oil Red O staining, with GC showing the strongest signal among the three (Figure 4B). Compared with the effects of these compounds, hSCFs treated with SG, KCED-1, or KCED-2 showed remarkably increased signals in lipid staining in dose-dependent manners, indicating the increased formation of lipid droplets and strong adipogenesis by the KCEDs. Consistent with the results observed with induction of APN mRNA and protein levels, KCED-2 showed the stronger effect in inducing adipogenesis than SG and KCED-1, when considering the signal intensity at identical concentrations.

### 2.4. APN-Containing Culture Supernatants Induce the Expression of Hair Growth-related Factors in Human Follicular Dermal Papilla Cells

**Figure 5:**
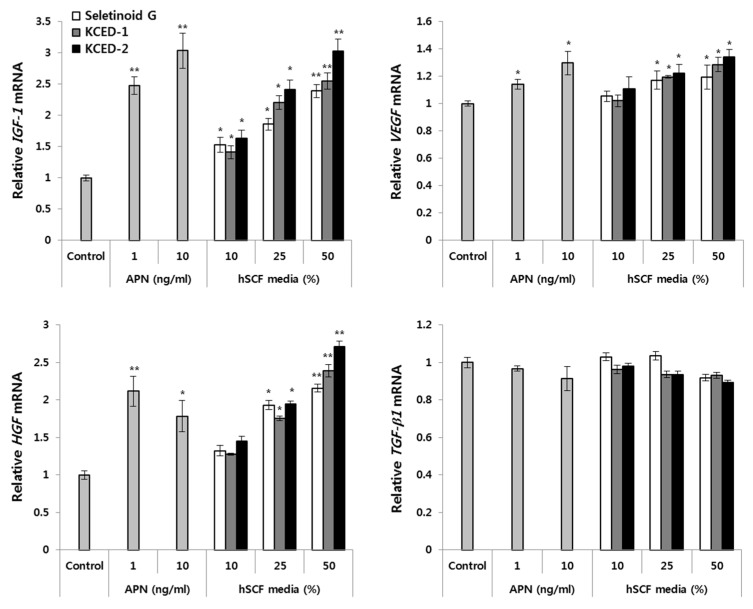
The SG-, KCED-1-, or KCED-2-treated hSCF media increase the mRNA levels of hair growth-related growth factors in hDPCs. The hDPCs were treated with different volumes of 50 μM SG-, KCED-1-, or KCED-2-treated hSCFs media for 48 h. The mRNA expression of hair growth-related growth factors was analyzed by RT-qPCR using respective Taqman probes. The data are presented as the mean ± SD (*n* = 3; * *p* < 0.05, ** *p* < 0.01).

Based on the previous report that APN shows hair growth-promoting effect on human hair follicles in vitro and ex vivo organ culture [9], APN has been suggested as a potential hair growth-promoting adipokine. Therefore, we investigated whether the hSCF-derived, APN-enriched conditioned media after treatments of SG, KCED-1, or KCED-2 could influence the expression of hair growth-related factors in hDPCs, which are known to express APN receptors [9]. Synthetic APN peptides were used as positive control and the treatment of these peptides significantly upregulated the mRNA expression of insulin-like growth factor 1 (*IGF-1*), vascular endothelial growth factor (*VEGF*), and hepatocyte growth factor (*HGF*), which were previously verified to be increased by APN in cultured hDPCs [9], compared with that seen in non-treated control (Figure 5). In contrast to these growth factors, transforming growth factor beta-1 (*TGF-β1*) was not significantly affected by APN peptides in our culture system, although it was reduced by APN treatment in hDPCs in previous study [9]. We treated hDPCs with various concentrations (5%, 10%, and 50%) of SG-, KCED-1-, or KCED-2-treated hSCF conditioned media for 48 h and examined the mRNA expression of hair growth-related factors. As expected, hDPCs showed increased mRNA levels of *IGF-1*, *VEGF*, and *HGF* in a concentration-dependent manner and the effects were comparable to those seen after APN peptide-treatment (Figure 5). *TGF-β1* mRNA levels were prone to be decreased weakly but relatively by the treatments of KCED-1-, KCED-2-treated, or 50% of SG-treated hSCF conditioned media compared with control treatment. These results suggest that SG-, KCED-1-, or KCED-2-treated hSCF media could contribute to hair growth promotion in vitro by stimulating hDPCs similar to treatment with APN. 

Based on the APN amounts released by treatment of hSCFs with 50 μM SG, KCED-1, or KCED-2 (Figure 4A), the APN concentration in hDPC culture medium treated with 50% hSCF-conditioned medium was estimated to be in the range of about 1.5 to 2.5 ng/mL, which was comparable to those of APN peptides effectively upregulating the expression of hair growth-related factors (Figure 5). Besides APN, other secretory factors might be induced by SG, KCED-1, or KCED-2 treatments in hSCFs and contribute to hair growth promotion in hDPCs. Whether the enriched APNs in SG-, KCED-1-, or KCED-2-treated hSCF media are mainly involved in inducing mRNA expression of hair growth-related factors in hSCFs remains to be addressed using neutralizing antibodies or siRNAs against APN receptors. At least, SG, KCED-1, and KCED-2 did not seem to affect hair growth-related factors by themselves in hDPCs based on our preliminary examination. 

**Figure 6:**
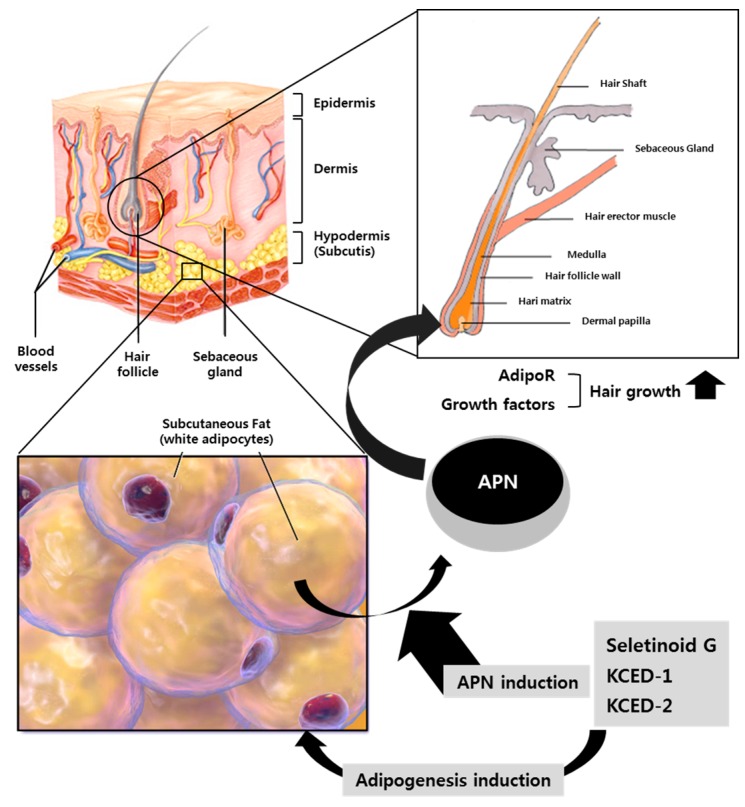
Schematic diagram illustrating the roles of kojyl cinnamate ester derivatives in adipogenesis and hair growth. Kojyl cinnamate ester derivatives including SG, KCED-1, and KCED-2 can promote adipogenesis and induction of *APN* mRNA levels and secretion of APN protein from human subcutaneous fat tissue. The secreted APN can promote hair growth via APN receptors.

There are several items of literature claiming that hair follicles (HFs) interact strongly with adipocytes in dermal white adipose tissue (dWAT) located in the superficial layer of the subcutaneous adipose tissue (sWAT) and that HF cycling correlates with spatiotemporal behavior of these adipocytes, i.e., it has different ratio of proliferation (preadipocyte) and differentiation (mature adipocyte) states [18,19,20,21], suggesting close interaction between HFs and dermal and subcutaneous WAT. Therefore, dermal adipocytes have been suggested as a potential target not only for counteracting skin aging but also for hair growth [21,22]. Interactions between HFs and adipocytes are expected to involve various soluble factors including microRNAs and possibly APN secreted from adipocytes from dermal and subcutaneous WAT. Extracellular vesicles containing exosome derived from skin adipocytes are also considered to be potent mediators reflecting local WAT contents, i.e., immature and mature states of adipocyte during the hair cycle [21]. Considering the three-dimensional structure of HF, which is surrounded by the epidermis, dermis, vessels, sebaceous gland, hair erector muscle, and dermal and subcutaneous WAT, the effects of APN and APN-enriched conditioned media derived from hSCFs on hair growth may be worthy of being evaluated via human HF organ culture. A schematic diagram for the roles of the kojyl cinnamate ester derivatives SG, KCED-1, and KCED-2 in adipogenesis and hair growth is illustrated in Figure 6. Given that intrinsic or extrinsic aging is known to correlate with a continuous reduction of WAT [22], we expect that kojyl cinnamate ester derivatives, as well as APN, could be also used as anti-aging agents by promoting adipogenesis of dermal and subcutaneous WAT.

## 3. Materials and Methods 

### 3.1. Compounds 

Seletinoid G [IUPAC name: (5-hydroxy-4-oxo-4H-pyran-2-yl)methyl (2E)-3-(2H-1,3-benzodioxol-5-yl)prop-2-enoate; Patent #, KR2016-0116831], kojyl cinnamate ester derivative-1 (KCED-1) [IUPAC name: 3-(3,4,5-Trimethoxy-phenyl)-acrylic acid 5-hydroxy-4-oxo-4*H*-pyran-2-ylmethyl ester] and KCED-2 [IUPAC name: 3-(2,6,6-Trimethyl-cyclohex-1-enyl)-acrylic acid 5-hydroxy-4-oxo-4H-pyran-2-ylmethyl ester] were synthesized and supplied by R&D Unit, Amorepacific Corp. (Yongin, South Korea). The structure of each compound is described in Figure 1. KA, CA, and GC were purchased from Sigma-Aldrich (St. Louis, MO, USA). Human adiponectin was purchased from ProSpec (Rehobot, Israel).

### 3.2. Cell Culture, Differentiation, and Compound Treatment

Human subcutaneous fat cells (hSCFs) representing subcutaneous preadipocytes (#SP-F-2) and Subcutaneous Preadipocyte Media were purchased from ZenBio Inc. (Research Triangle Park, NC, USA) and cultured in a humidified 5% CO_2_ incubator. To induce differentiation, hSCFs in a confluency ≥95% were cultured in Dulbecco’s modified Eagle’s medium (DMEM; Lonza, Walkersville, MD, USA) supplemented with 10% fetal bovine serum (FBS; PAA, Pasching, Austria), 10 µg/mL insulin (Sigma-Aldrich, St. Louis, MO, USA), 0.5 mM 3-isobutyl-1-methylxanthine (IBMX; Sigma-Aldrich, St. Louis, MO, USA), 1 µM dexamethasone (DEX; Sigma-Aldrich, St. Louis, MO, USA), and 1 µM troglitazone (Sigma-Aldrich, St. Louis, MO, USA) for two days. The cells were further incubated in DMEM supplemented with 10% FBS and 10 μg/mL insulin with or without compounds for additional 14 days. The medium containing compounds was replaced every other day. Human follicular dermal papilla cells (hDPCs) and the culture medium were purchased from Cefobio Co. (Seoul, Korea). The hDPCs were cultured in a humidified 5% CO_2_ incubator according to the manufacturer’s instruction.

### 3.3. Cell Viability Assay

The hSCF viability was measured using EZ-Cytox Cell viability assay kit (MTT assay, Daeil lab Service, South Korea) according to the manufacturer’s instructions. In brief, hSCFs were cultured for seven days and treated with various concentrations of each chemical (SG, KCED-1, KCED-2) for 24 and 72 h. EZ-Cytox solution (10 μL) was added to each well and incubated at 37 °C for 2 h. Absorbance at 450 nm was measured using a spectrophotometer (Synergy H2, BioTek., Winooski, VT, USA). All experiments were triplicated and the data are presented as the absorbance.

### 3.4. Quantitative Real-time PCR (RT-qPCR)

Total RNA was extracted using TRIzol reagent (Life Technologies, Carlsbad, CA, USA) according to the manufacturer’s instructions. One μg of total RNA was utilized to synthesize cDNAs using the RevertAid First Strand cDNA Synthesis kit (Thermo Scientific, Waltham, MA, USA). One μg of cDNA sample was subjected to PCR analysis using each TaqMan^®^ probe (Life Technologies, Carlsbad, CA, USA), Quantitect Probe PCR kit (Qiagen, Valencia, CA, USA), and the 7500 fast real-time PCR system (Life Technologies, Carlsbad, CA, USA). Each TaqMan^®^ probe was as follows: Adiponectin (*ADIPOQ*; #Hs00605917_m1), insulin-like growth factor 1 (*IGF-1*; #Hs01547656_m1), vascular endothelial growth factor (*VEGF*; #Hs00900055_m1), hepatocyte growth factor (*HGF*; #Hs00300159_m1), transforming growth factor β-1 (*TGF-β1*; #Hs00998133_m1), and glyceraldehyde-3-phosphate dehydrogenase (*GAPDH*; #4352339E). All data were acquired from three independent experiments and are presented as a fold change relative to the *GAPDH* control.

### 3.5. ELISA Assay for Secreted Adiponectin

The hSCFs were treated with various concentrations of each chemical (SG, KCED-1, and KCED-2) and differentiated for 14 days. The culture medium was collected and centrifuged at 13,000 rpm for 15 min to remove any debris. The secreted adiponectin was measured using adiponectin ELISA kit (Enzo Life Sciences, Farmingdale, NY, USA), following the manufacturer’s instructions.

### 3.6. Oil Red O Staining

The differentiated hSCFs were washed twice with cold PBS and fixed with 3.7% formaldehyde (Sigma-Aldrich, St. Louis, MO, USA) for 1 h. The fixed cells were washed with 60% propylene glycol (Sigma-Aldrich, St. Louis, MO, USA) in PBS and were stained with a working solution of Oil Red O (ORO; 0.3% ORO in 60% propylene glycol; Sigma-Aldrich, St. Louis, MO, USA) for 30 min. The cells were washed with 85% propylene glycol thrice and rinsed with tap water. Lipid droplets stained with ORO dye were visualized with an IX71 microscope (Olympus, Tokyo, Japan).

### 3.7. Statistical Analysis

All data are presented as the mean ± SD. Two-tailed Student’s *t*-tests were used to analyze the differences between pairs of groups, and the threshold for statistical significance was set at 0.05 (* *p* < 0.05). Multiple groups were analyzed with one-way ANOVA.

## 4. Conclusions

We demonstrated that the kojyl cinnamate ester derivatives including SG, KCED-1, and KCED-2 induce APN production in vitro and stimulate adipogenesis in hSCFs. In addition, SG-, KCED-1-, or KCED-2-treated hSCF media increased the mRNA levels of hair growth-related growth factors in hDPCs. Based on our observations, we propose that SG, KCED-1, and KCED-2 are potent APN inducers and could be used in cosmetic and dermatological products for regulating adipogenesis in subcutaneous fat tissue and for promoting hair growth through stimulation of APN-associated hair growth signaling.

## Abbreviations

APN	Adiponectin
KCED	Kojyl cinnamate ester derivative
hSCFs	Human subcutaneous fat cells
AdioR	Adiponectin receptor
hDPCs	Human follicular dermal papilla cells
SG	Seletinoid G
ADSCs	Adipose tissue-derived stem cells
KA	Kojic acid
CA	Cinnamic acid
GC	Glibenclamide
IGF-1	Insulin-like growth factor 1
VEGF	Vascular endothelial growth factor
HGF	Hepatocyte growth factor
TGF-β1	Transforming growth factor β-1
HF	Hair follicle
dWAT	Dermal white adipose tissue
sWAT	Subcutaneous white adipose tissue
